# Associations of the Cardiometabolic Index with the Risk of Cardiovascular Disease in Patients with Hypertension and Obstructive Sleep Apnea: Results of a Longitudinal Cohort Study

**DOI:** 10.1155/2022/4914791

**Published:** 2022-06-23

**Authors:** Xintian Cai, Junli Hu, Wen Wen, Jingyu Wang, Mengru Wang, Shasha Liu, Qing Zhu, Jing Hong, Yujie Dang, Xiaoguang Yao, Le Sun, Delian Zhang, Qin Luo, Nanfang Li

**Affiliations:** ^1^Hypertension Center of People's Hospital of Xinjiang Uygur Autonomous Region, Xinjiang Hypertension Institute, National Health Committee Key Laboratory of Hypertension Clinical Research, Key Laboratory of Xinjiang Uygur Autonomous Region, Xinjiang Clinical Medical Research Center for Hypertension Diseases, Urumqi, China; ^2^Renal Division, Peking University First Hospital, Beijing, China

## Abstract

**Purpose:**

We aimed to explore the relationship between the cardiometabolic index (CMI) and cardiovascular disease (CVD) and its subtypes (coronary artery disease and stroke) in patients with hypertension and obstructive sleep apnea (OSA).

**Methods:**

We conducted a retrospective cohort study enrolling 2067 participants from the Urumqi Research on Sleep Apnea and Hypertension study. The CMI was calculated as triglyceride to high‐density lipoprotein cholesterol ratio × waist‐to‐height ratio. Participants were divided into three groups (T1, T2, and T3) according to the tertile of CMI. The Kaplan-Meier method helped to calculate the cumulative incidence of CVD in different groups. We assessed the association of CMI with the risk of CVD and CVD subtypes by estimating hazard ratios (HRs) and 95% confidence intervals (CIs) using Cox models.

**Results:**

During a median follow-up of 6.83 years (interquartile range: 5.92-8.00 years), 326 incident CVD were identified, including 121 incident stroke and 205 incident coronary heart disease (CHD). Overall, after adjusting for confounding variables, CMI was positively associated with the risk of new-onset CVD (per SD increment, adjusted HR: 1.31; 95% CI: 1.20, 1.43), new-onset CHD (per SD increment, adjusted HR: 1.33; 95% CI: 1.20, 1.48), and new-onset stroke (per SD increment, adjusted HR: 1.27; 95% CI: 1.10, 1.47). Similar results were obtained in various subgroup and sensitivity analyses. Adding CMI to the baseline risk model for CVD improved the *C*-index (*P* < 0.001), continuous net reclassification improvement (*P* < 0.001), and integrated discrimination index (*P* < 0.001). Similar results were observed for CHD and stroke.

**Conclusion:**

There was a positive association between CMI levels and the risk of new-onset CVD in patients with hypertension and OSA. This finding suggests that CMI may help identify people at high risk of developing CVD.

## 1. Introduction

According to epidemiological studies, cardiovascular disease (CVD) has been the leading cause of morbidity and mortality of chronic noncommunicable diseases worldwide, with coronary heart disease (CHD) and stroke being the leading causes of CVD [1]. In 2016, there were more than 290 million cases of CVD in China, including 1.736 million deaths from coronary heart disease and 4.344 million deaths from stroke, causing a profound socioeconomic burden on society [2, 3]. Currently, both hypertension and obstructive sleep apnea (OSA) are considered to be significant independent risk factors for CVD [4–8]. Furthermore, the coexistence of OSA and hypertension further increases the risk of CVD [9–11]. Therefore, there is a requirement to identify hypertensive patients with OSA who are at high risk for CVD to provide more cost-effective care and facilitate service policy planning.

Epidemiological and pathophysiological studies have shown that the accumulation of visceral adipose tissue (VAT) is associated with a variety of CVD risk factors, including type 2 diabetes, hypertension, and metabolic syndrome [12–16]. Nowadays, imaging techniques, such as computed tomography, dual-energy X-ray absorptiometry, and magnetic resonance imaging, can accurately quantify the volume of VAT as well as the distribution of specific compartments [16–19]. However, these methods are not feasible in routine physical examinations because they are time-consuming and expensive and have radiation risks.

The cardiometabolic index (CMI), a recently developed index reflecting VAT distribution and dysfunction, is useful in assessing the risk of obesity-related metabolic diseases such as diabetes, hypertension, left ventricular hypertrophy, and stroke [20–25]. CMI consists of anthropometric and biochemical indicators, including waist-to-height ratio (WHtR) and triglyceride to high-density lipoprotein cholesterol ratio (TG/HDL-C), which can be easily obtained during a health examination [26]. WHtR is considered a valuable parameter that reflects the function of subcutaneous adipose tissue (SAT) and VAT compartments [27–29]. TG/HDL-C is a simple marker that has been considered a good indicator of the risk of CVD and can closely reflect dyslipidemia and insulin resistance (IR) [30–32].

To our knowledge, there are few studies on the relationship between CMI and the risk of CVD, especially in hypertensive patients with OSA. Therefore, this study is aimed at investigating the relationship between CMI and the risk of CVD and its subtypes using data from the Urumqi Research on Sleep Apnea and Hypertension (UROSAH) study.

## 2. Methods

### 2.1. Study Population

**Figure 1:**
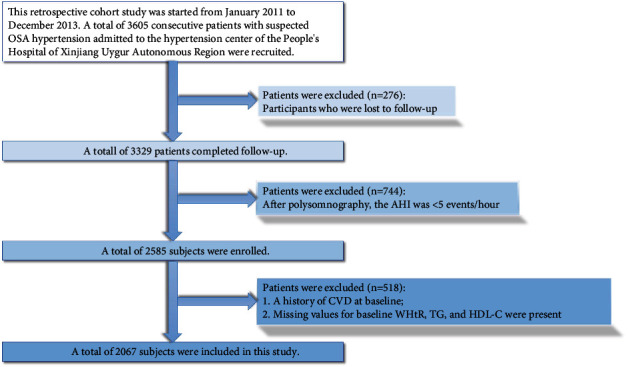
Flow chart of study participant selection.

All data were obtained from the UROSAH study, and a detailed description of the UROSAH study has been reported elsewhere [33]. In brief, UROSAH is a single-center, observational, retrospective cohort study designed to investigate the relationship between long-term cardiovascular outcomes in patients with hypertension and OSA. In this study, the study population was hypertensive patients diagnosed with OSA at the Hypertension Center of Xinjiang Uygur Autonomous Region People's Hospital between January 2011 and December 2013. Patients with a definite prior diagnosis of hypertension or with a newly diagnosed hypertension at admission, criteria of which were referred to guidelines from the Chinese Hypertension League, were considered to have hypertension. All patients were diagnosed with OSA after admission by laboratory nocturnal polysomnography (PSG), which was defined in detail following the relevant guidelines. Other inclusions and exclusion criteria are detailed in the flow chart (Figure 1). Overall, 2067 participants were ultimately included in this analysis after strict adherence to the inclusion and exclusion criteria.

### 2.2. Ethical Approval

The ethics application was approved by the Ethics Committee of the Xinjiang Uygur Autonomous Region People's Hospital (reference: 2019030662). All patients gave informed consent according to the Declaration of Helsinki.

### 2.3. Assessment of Covariates

Baseline data collection was expected to be completed by professional research staff following the operational procedures mentioned in previous studies [33]. One-on-one interviews were held with each participant using a standardized electronic medical record. Diabetes was defined as fasting glucose ≥ 7.0 mmol/L, use of any glucose-lowering medication, or self-reported history of diabetes. Regular continuous positive airway pressure (CPAP) treatment was defined as the use of CPAP therapy for more than 70% of nights throughout the follow-up period and no less than 4 hours per night, or an average of ≥4 hours per night [34, 35]. Similar to the definition of CPAP treatment use, subjects with at least 4 hours of use per night and at least 70% of nights of continuous use were considered to be regular oral appliance treatment users [36].

### 2.4. Laboratory Assays

On the morning of the survey, venous blood samples were harvested after an overnight fast (>8 hours) and sent to the central laboratory for analysis shortly after sampling. All biochemical measurements including total cholesterol (TC), TG, HDL-C, low-density lipoprotein cholesterol (LDL-C), and fasting plasma glucose (FPG) were measured on a fully automated biochemical analyzer (Hitachi 7600; Japan). The estimated glomerular filtration rate (eGFR) was derived by using the equations of the Chronic Kidney Disease Epidemiology Collaborative Group [37].

### 2.5. Calculation of CMI

The TG/HDL-C ratio was computed by dividing the serum concentration of TG (mmol/L) by HDL-C (mmol/L). WHtR was derived by dividing WC (cm) by height (cm). CMI was computed as TG/HDL‐C × WHtR according to the formula in previously published papers [26].

### 2.6. OSA Evaluation

All participants underwent nocturnal PSG monitoring in the sleep laboratory. All sleep monitoring results were analyzed by specialized sleep technicians according to criteria [38, 39]. Details of the PSG and scoring criteria applied in this research are available in the Supplementary Material. OSA was defined as an apnea-hypopnea index (AHI) of ≥5 events/h. The severity of OSA was classified as normal (<5), mild (5-14.9), moderate (15-29.9), and severe (≥30) according to the AHI [39, 40].

### 2.7. Endpoints and Follow-Up

In this study, the primary outcome was new-onset cardiovascular disease (CVD), including stroke and coronary heart disease (CHD). Cardiologists from the Clinical Events Committee of a tertiary care hospital diagnose CVD after reviewing all medical records. CHD was defined as myocardial infarction (fatal or nonfatal), coronary revascularization (percutaneous transluminal coronary angioplasty or coronary artery bypass grafting), and angina pectoris (unstable or stable), which were diagnosed by electrocardiogram, signs or symptoms of ischemia, or diagnostic enzymes. A stroke was defined as a sudden or rapid onset of focal neurological deficit lasting longer than 24 hours or until death. The diagnosis was confirmed by computed tomography scan, magnetic resonance imaging, or the patient's clinical symptoms. Stroke includes both ischemic and hemorrhagic strokes. After entry into this cohort, subjects were followed up for the first occurrence of CVD. Follow-up results were available through outpatient interviews, telephone interviews, and inpatient medical records. Deaths due to CVD were verified by hospital death certificates or hospitalization data or by the local police system. The follow-up period began with the initial visit and continued until the participant had a new onset of CVD or died (whichever occurred first), otherwise until the end of the follow-up period in January 2021.

### 2.8. Statistical Analysis

Means ± standard deviation (SD), medians (Q25, Q75), or proportions were illustrated for characteristics of the participants by CMI tertiles. Differences in group characteristics were compared by using Fisher's exact test, ANOVA test, Kruskal-Wallis test, or *χ*^2^ test, accordingly. Kaplan-Meier curves and log-rank tests were used to present the unadjusted cumulative hazards across the tertiles of CMI.

Multicollinearity among the variables was tested by calculating the variance inflation factor (VIF). Variables with VIF > 5 were considered to have severe multicollinearity and could not be incorporated in the multivariate regression analysis. Hazard ratios (HRs) and 95% confidence intervals (CIs) of new-onset CVD or its subtypes (CHD and stroke) in relation to CMI (continuous, tertiles, and categories) were calculated through the use of Cox proportional hazards models. Model 1 was adjusted for age and sex; Model 2 was further adjusted for drinking status, history of diabetes, smoking status, DBP, SBP, and BMI; Model 3 was further adjusted for all noncollinear variables. After entering the median CMI values of each tertile into the model and treating them as continuous variables, the regression model was tested for trend. To test the robustness of our results, we performed several sensitivity analyses. First, we excluded participants who were current smokers and alcohol drinkers and repeated the analysis. Second, we excluded participants who were regularly receiving CPAP treatment. Third, we excluded events that occurred one year prior to follow-up to minimize potential reverse causes. Finally, non-CVD deaths were considered a competing risk, and we performed a competing risk regression analysis based on the methods reported by Fine and Gray. Furthermore, we explored the relationship between the CMI and new-onset CVD, CHD, and stroke using a generalized additive model and smoothed curve fitting.

Stratified analyses and interactions were separately performed by age (<60 or ≥60years), sex (female or male), SBP (<140 or ≥140 mmHg), BMI (<28 or ≥28 kg/m^2^), smoking (never, past, or current), DBP (<90 or ≥90 mmHg), drinking (never, past, or current), and AHI (≥30, ≥15 to <30, or ≥5 to <15 events/h).

Receiver operating characteristic (ROC) curves were used to estimate the predictive values of possible predictors for endpoints (CVD, CHD, and stroke). The *C*-index, continuous net reclassification improvement (NRI), and integrated discrimination index (IDI) were used to evaluate the additional predictive value of the CMI beyond established risk factors. Confidence intervals for *C*-index, continuous NRI, and IDI were estimated using 1000 bootstrap procedures.

All analyses were undertaken with R software (version 4.0.1). The threshold of significance was fixed at 5% (*P* < 0.05 was significant).

## 3. Results

### 3.1. Baseline Characteristics of Participants

A total of 2067 eligible subjects with a mean age of 49.51 ± 10.73 years were included in this study, of which 68.92% were male. Baseline characteristics of subjects according to CMI tertile are presented in Table 1. Participants in the other groups were more likely to be younger, male, more current smokers, and drinkers; have a higher prevalence of diabetes; and have a higher BMI, eGFR, TC, TG, FPG, and AHI levels and a lower HDL-C level compared to the tertile 1 group.

### 3.2. Association between CMI and Risk of CVD and Its Subtypes

**Figure 2:**
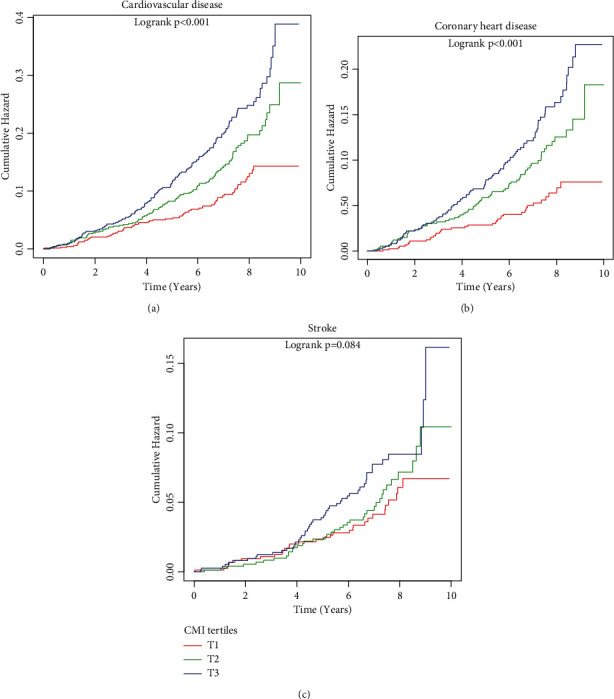
Cumulative hazards of risk of (a) cardiovascular disease, (b) coronary heart disease, and (c) stroke according to the tertile of the cardiometabolic index.

During a median follow-up of 6.83 years (interquartile range: 5.92-8.00 years), 326 (14.19%) incident CVD were identified, including 121 (5.27%) incident stroke and 205 (8.92%) incident CHD. Furthermore, cumulative hazard curves of cardiovascular events across CMI tertiles are shown in Figure 2 (log-rank test: *P* < 0.001 for CVD and CHD, respectively; *P* = 0.084 for stroke).

**Figure 3:**
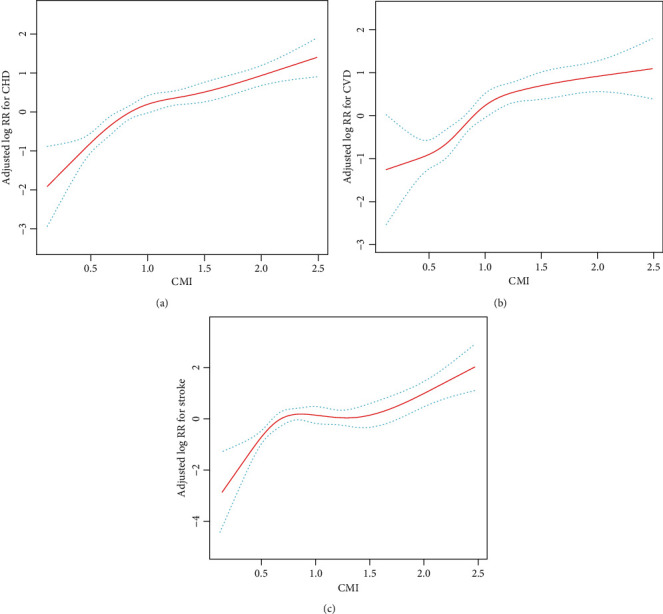
Smoothing curve revealing the effect of the cardiometabolic index on the risk of (a) cardiovascular disease, (b) coronary heart disease, and (c) stroke. ^∗^Adjusted for all noncollinear variables.

In total, there was a positive relation between CMI and the risk of new-onset CVD (Figure 3(a)), new-onset CHD (Figure 3(b)), and new-onset stroke (Figure 3(c)). As presented in Table 2, the HR (95% CI) for CVD in the fully adjusted regression model was 1.31 (1.20, 1.43) for per SD increase in CMI. Fully adjusted HRs (95% CIs) for CVD were 1.60 (1.18, 2.16) and 2.17 (1.63, 2.90) for subjects in tertile 2 and tertile 3, respectively. The risk of CVD increased substantially from quartile 1 to quartile 3 (*P* for trend < 0.001). Similarly, there was a meaningful association between CMI and CHD and stroke in the fully adjusted regression model (Table 2). For each SD increase in the CMI, the HR (95% CI) of the CHD rate was 1.33 (1.20, 1.48) in the fully adjusted regression model. The fully adjusted HR (95% CI) for CHD in tertile 3 was 2.62 (1.79, 3.83). The increased risk of CHD from tertile 1 to tertile 3 was statistically significant (*P* for trend < 0.001). Also, a remarkable positive association was shown between CMI and stroke (per SD increment; HR: 1.27; 95% CI: 1.10, 1.47). In the fully adjusted regression model, the HRs (95% CIs) for stroke were 1.23 (0.77, 1.98) and 1.64 (1.05, 2.57) for participants in tertile 2 and tertile 3, respectively, compared with participants in tertile 1 (*P* for trend = 0.029). In addition, the risk of new-onset CVD (HR: 1.68; 95% CI: 1.35, 2.09), CHD (HR: 1.81; 95% CI: 1.37, 2.39), and stroke (HR: 1.47; 95% CI: 1.02, 2.12) was significantly higher in fully adjusted regression models for participants in tertile 3 of CMI level (≥1.21) compared with those in tertile 1-2 (<1.21) (Table 2).

### 3.3. Sensitivity Analysis

In sensitivity analyses, the association between CMI and the risk of CVD, CHD, and stroke did not change substantially after excluding participants who were regularly treated with CPAP (Supplemental Table 2), or after excluding participants who were current smokers and drinkers (Supplemental Table 3 and Supplemental Table 4), or after excluding participants who developed CVD within the first year of follow-up (Supplemental Table 5). In addition, a sensitivity analysis was performed using the Fine-Gray model to reduce the effect of competing mortality risks. The results of the competing risks model were similar to those of the Cox model (Supplemental Table 6).

### 3.4. Subgroup Analysis

**Figure 4:**
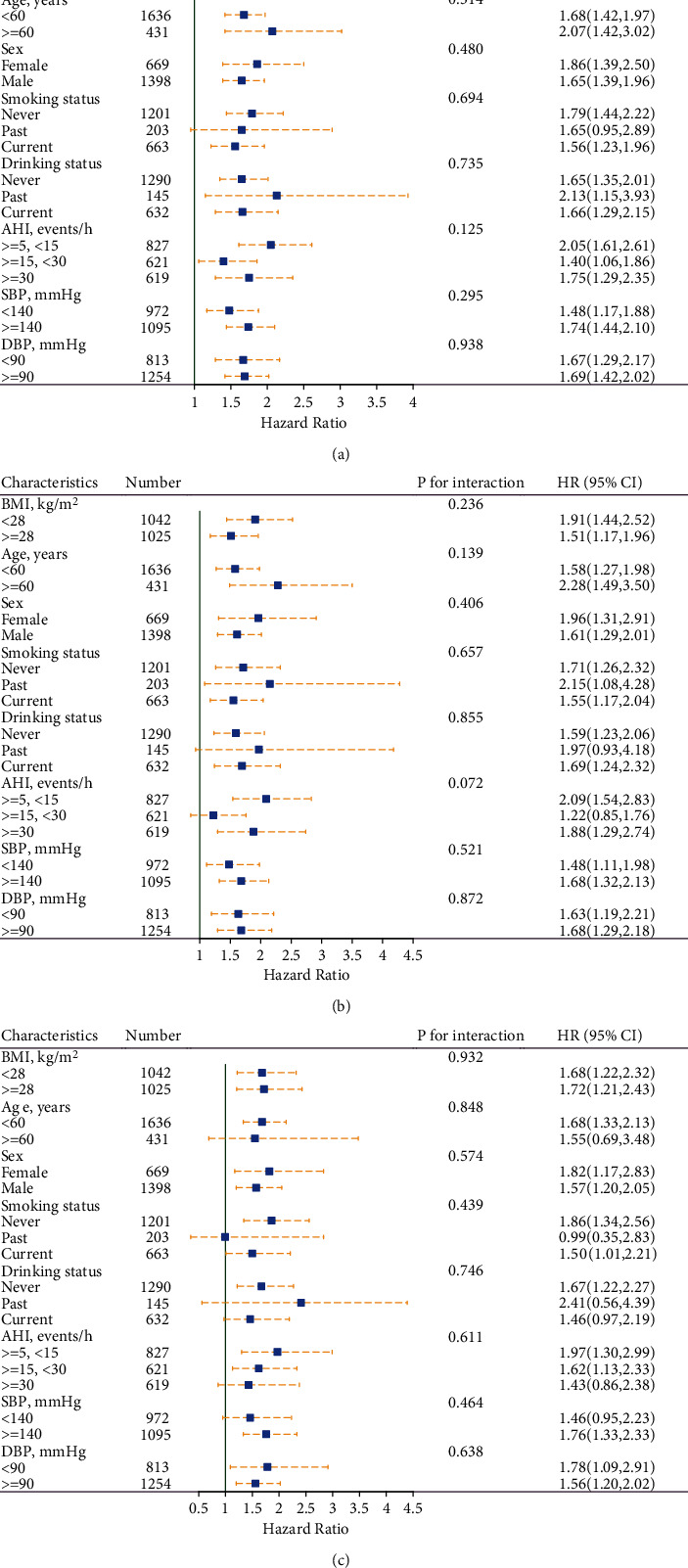
Association of the cardiometabolic index (per SD increment) with risk of (a) cardiovascular disease, (b) coronary heart disease, and (c) stroke in various subgroups. ^∗^Adjusted for all noncollinear variables, if not be stratified.

We further performed a stratified analysis to assess the association of CMI (per SD increment) with risk of CVD (Figure 4(a)), risk of CHD (Figure 4(b)), and risk of stroke (Figure 4(c)). None of the variables, including age (<60 vs. ≥60 years), sex (female vs. male), SBP (<140 vs. ≥140 mmHg), BMI (<28 vs. ≥28 kg/m^2^), smoking (never vs. past vs. current), DBP (<90 vs. ≥90 mmHg), drinking (never vs. past vs. current), and AHI (≥ 30 vs. ≥ 15 to <30 vs. ≥ 5 to <15 events/h), were found to modify the association between CMI (per SD increment) and the risk of CVD (all *P* interactions > 0.05). Similar outcomes were observed for CHD and stroke.

### 3.5. ROC Curve Analysis of Different Markers

The results of the ROC analysis for CMI and other predictors are shown in Table 3 and Supplementary Figure 1. Compared to TC, TG, HDL-C, TG/HDL-C, LDL-C, FPG, and WHtR, CMI was the strongest predictor of CVD (AUC = 0.617), with a sensitivity of 0.675, a specificity of 0.509, and the best threshold of 1.006 (Table 3). Similar outcomes were observed for CHD and stroke.

### 3.6. Additive Effect of CMI to Established Risk Factors

The additional value of CMI for risk prediction of CVD, CHD, and stroke was evaluated based on discrimination (*C*-index), continuous NRI, and IDI (Table 4). The addition of CMI significantly improved the reclassification and discrimination for CVD prediction over the baseline risk model with a continuous NRI of 0.179 (0.114, 0.248) and an IDI of 0.013 (0.005, 0.025) (both *P* < 0.001). Similarly, the reclassification and discrimination for CHD and stroke prediction were also significantly improved with the addition of CMI. The *C*-index for predicting CVD, CHD, and stroke without CMI in the established risk factors were 0.600 (0.567, 0.633), 0.639 (0.602, 0.677), and 0.540 (0.484, 0.596), respectively. However, the addition of CMI to the established risk factors significantly improved the prediction of new-onset CVD, new-onset CHD and new-onset stroke with *C*-index of 0.648 (0.615, 0.681), 0.675 (0.638, 0.712), and 0.596 (0.541, 0.652), respectively.

## 4. Discussion

Despite tremendous efforts to improve clinical outcomes in recent decades, CVD is still the leading cause of mortality and morbidity in China [41, 42]. The purpose of this study was to evaluate the relationship between CMI and the risk of CVD events. In this retrospective cohort study for the UROSAH study, we concluded that there was a significant association between CMI and the risk of CVD. Notably, the risk of CVD increased with increasing CMI during a median follow-up of 6.83 years. A similar pattern was found in CHD and stroke. After adjusting for possible confounders, the risk of CVD, CHD, and stroke was 2.17, 2.62, and 1.64 times higher in the group with the highest CMI level than in the lowest group. With each SD increase in CMI level, the risk of CVD increased by 31%, the risk of CHD increased by 33%, and the risk of stroke increased by 27%. These trends persisted when multiple sensitivity analyses and subgroup analyses were performed, indicating that the relationship is quite robust. Furthermore, prevention strategies that allow for early lifestyle interventions are a critical solution to reducing the burden of CVD [43]. Several guidelines recommend aggressive lifestyle interventions, such as limiting saturated fat intake, weight loss, and regular exercise, to lower CVD risk [44–46]. However, sustainable implementation of lifestyle interventions has long faced several challenges and cannot be overcome by a one-size-fits-all approach. Instead, sustainable implementation of lifestyle interventions requires, in particular, individualized lifestyle advice [47]. To be able to provide targeted lifestyle advice, it is paramount to adequately risk stratify individuals in preclinical status. In this study, the addition of CMI to a baseline risk model that includes traditional risk factors significantly enhances the ability to stratify risk. Our findings facilitate the identification of associations between CMI and CVD events in hypertensive patients with OSA and the screening of high-risk groups. In this way, medical professionals can develop appropriate prevention strategies for high-risk populations.

Obesity is a well-recognized risk factor for CVD [48]. However, several epidemiological surveys have revealed a protective effect of obesity on CVD outcomes classified by BMI and have questioned the nature of the relationship between obesity and CVD [49–51]. This paradox, also known as the “obesity paradox” or “BMI paradox,” suggests that BMI or other general measures of adiposity do not assess the actual metabolic status and body fat distribution [13, 52]. Compared to general adiposity, VAT may better reflect the state of natural metabolic impairment in obesity [13]. VAT is a complex, metabolically active tissue that produces different adipokines and hormones and leads to endocrine-metabolic comorbidities [53, 54]. It is also closely associated with increased adipocytokine production, proinflammatory activity, and disturbed lipid levels [51, 55, 56]. The accumulation of VAT determines cardiometabolic risk and cardiometabolic-related mortality, rather than the degree of obesity in general [12]. Currently, most measurements of VAT are performed by dual-energy X-ray absorptiometry, computed tomography, or magnetic resonance tomography. However, these imaging techniques are not appropriate for routine examinations because of their time expenditure, high cost, and radiation hazard [17, 18, 57].

CMI is a fresh index that was first proposed by Wakabayashi and Daimon in 2015 and was presumed to have significant advantages in the assessment of diabetes mellitus [26]. Subsequent studies further found that CMI could be an ideal marker for identifying VAT distribution and associated metabolic dysfunction and showed a close association with VAT measured with techniques such as magnetic resonance imaging [26, 58]. Several studies have shown that CMI is a useful screening tool for different populations, identifying those with worsening metabolic features and a higher risk of CVD, such as abnormal left ventricular geometry, arterial stiffness, hyperuricemia, diabetes, hypertension, and ischemic stroke [22, 24, 25, 59, 60]. One of the components of CMI, WHtR, is an alternative tool to BMI or WC to identify individuals at higher metabolic risk. WHtR has been reported to be a better discriminator of CHD and stroke risk than WC and BMI [61–63]. The TG/HDL-C ratio is likewise considered a decent indicator of CVD risk [64, 65]. The TG/HDL-C ratio has been shown to reflect small atherogenic LDL-C particles and is strongly associated with IR and metabolic syndrome [65–67]. Metabolic syndrome and IR are well-known markers of CVD risk, especially diabetes, hypertension, hyperlipidemia, and atherosclerosis [68, 69].

The mechanisms underlying the association between CMI and CVD and its subtypes are unclear. CMI is a surrogate marker for VAT and may primarily explain these associations. First, VAT has a high lipolytic activity, leading to an increase in circulating free fatty acids, which promotes endothelial dysfunction [70, 71]. Due to its anatomical location, VAT drains through the portal vein, which can lead to increased secretion of free fatty acids and proinflammatory cytokines in the liver, which further may promote increased secretion of IR and very-low-density lipoproteins [70–72]. Second, increased VAT leads to adipose dysfunction and induces chronic local inflammation [73, 74]. The classical features of the inflammatory process due to VAT are infiltration of M1 macrophages, production of reactive oxygen species, and release of proinflammatory cytokines [75]. The chronic inflammatory environment is associated with the development of type 2 diabetes, metabolic syndrome, dyslipidemia, and hypertension, all of which are associated with an increased risk of cardiovascular events [76–78]. The rapid expansion of VAT volume leads to cellular hypoxia activating hypoxia-inducible factor 1-alpha, increasing expression of interleukin 6 and leptin, reducing lipocalin production, and mediating macrophage attraction to adipose tissue [79–81]. Increased leakage of free fatty acids (FFA) from hypertrophic adipocytes has been reported due to enhanced basal lipolysis [1]. The released FFA promotes inflammation by binding to toll-like receptors 2 and 4, thereby activating the nuclear factor-kappa B and c-Jun N-terminal kinase signaling pathways [54, 82]. Activation of the above pathways increases the synthesis and secretion of proinflammatory cytokines, which mediate most of the complications associated with atherosclerosis. Third, VAT is associated with an increase in plasma plasminogen activator or inhibitor-1, which also contributes to thrombosis during atherosclerosis [70, 71]. Fourth, VAT is also associated with metabolic dysfunction and an increased risk of IR and hyperglycemia [83]. Several epidemiological studies have shown that IR and hyperinsulinemia are more important risk factors for cardiovascular disease than obesity per se [68, 84]. In terms of potential biological mechanisms, it has been proposed that high levels of cytokines and low levels of lipocalin may interfere with glucose homeostasis and contribute to chronic hyperinsulinemia and IR [85]. Additionally, the rate of lipolysis in VAT is higher than in subcutaneous adipose tissue, increasing the circulation of nonesterified fatty acids, which may affect hepatic insulin inactivation and contribute to IR and hyperinsulinemia [86]. In addition, IR decreases eNOS-active adipose tissue and NO production, which leads to plaque inflammation [85, 87]. These mechanisms sensitize plaque rupture, which leads to the development of CVD.

Our study has several strengths. According to a previous study in Japan, CMI may be the most effective predictor of CVD after adjusting for confounders [88]. However, because the participants in the previous study were a community healthy population, all participants in the present study were hypertensive patients with OSA. Therefore, a most important finding is that our study is the first cohort study to reveal the association of CMI with CVD, including stroke and CHD, in hypertensive patients with OSA. Second, the loss to follow-up was low and the high response rate of participants in this study compared to previous studies and adjustment for more potential confounders. Third, the analysis of effect modification factors for different subgroups in this study yields better data utilization and more robust conclusions. Finally, our results of the primary analyses were similar in several sensitivity analyses, which suggests that the results are robust to different assumptions. However, some limitations of this study should be acknowledged. First, since this was a retrospective study, we could not determine a causal relationship between CMI and the risk of CVD. For this reason, we performed sensitivity analyses after excluding participants who developed CVD within 1 year and found consistent results. Second, lifestyle, psychosocial factors, and ethnic factors are known confounders of CVD risk factors, and without these data, we were unable to assess these potential confounders. Third, the results may be biased because we did not consider antiplatelet and lipid-lowering therapy's type, intensity, and variability. However, we adjusted for the use of these agents. We will further optimize the study design, enhance the follow-up of the cohort, and collect more comprehensive information to make the findings more generalizable and convincing. Finally, the fact that these results are from a single center limits the generalizability of our results to other populations.

## 5. Conclusion

In conclusion, CMI is an independent predictor of CVD and its subtypes in patients with hypertension and OSA. As a clinically reliable and convenient marker of VAT, CMI has great potential use in predicting and preventing CVD.

## Figures and Tables

**Table 1:** Characteristics among patients, stratified by CMI.

Characteristics	Tertile 1 (<0.73)	Tertile 2 (≥0.73 to <1.21)	Tertile 3 (≥1.21)	*P* value
*N*	689	689	689	
Age (years)	52.13 ± 11.41	49.05 ± 10.87	47.52 ± 9.43	<0.001
Male, *n* (%)	387 (56.17%)	480 (69.67%)	521 (75.62%)	<0.001
History of diabetes, *n* (%)	88 (12.77%)	115 (16.69%)	141 (20.46%)	<0.001
Smoking status, *n* (%)				<0.001
Never	491 (71.26%)	382 (55.44%)	347 (50.36%)	
Past	64 (9.29%)	73 (10.60%)	70 (10.16%)	
Current	134 (19.45%)	234 (33.96%)	272 (39.48%)	
Drinking status, *n* (%)				<0.001
Never	497 (72.13%)	393 (57.04%)	383 (55.59%)	
Past	28 (4.06%)	65 (9.43%)	54 (7.84%)	
Current	164 (23.80%)	231 (33.53%)	252 (36.57%)	
BMI (kg/m^2^)	27.21 ± 3.69	28.64 ± 3.90	29.39 ± 3.59	<0.001
SBP (mmHg)	140.05 ± 19.40	140.28 ± 20.60	139.42 ± 18.90	0.675
DBP (mmHg)	90.45 ± 13.85	91.84 ± 14.32	92.97 ± 13.74	0.002
eGFR (mL/min/1.73 m^2^)	94.05 ± 19.10	96.75 ± 21.67	97.26 ± 23.11	0.007
TC (mmol/L)	4.42 ± 1.34	4.56 ± 1.15	4.66 ± 1.04	<0.001
TG (mmol/L)	1.14 ± 0.32	1.79 ± 0.43	3.22 ± 1.35	<0.001
HDL-C (mmol/L)	1.32 ± 0.31	1.07 ± 0.21	0.93 ± 0.19	<0.001
LDL-C (mmol/L)	2.59 ± 0.79	2.78 ± 0.80	2.58 ± 0.77	<0.001
FPG (mmol/L)	5.06 ± 1.12	5.24 ± 1.22	5.55 ± 1.88	<0.001
AHI (events/h)	16.20 (9.30-29.35)	18.10 (10.30-34.15)	20.80 (11.20-37.02)	<0.001
Medication use, *n* (%)				
ACEIs/ARBs	302 (43.83%)	330 (47.90%)	317 (46.01%)	0.317
*β*-Blockers	126 (18.29%)	123 (17.85%)	137 (19.88%)	0.595
CCBs	396 (57.47%)	403 (58.49%)	443 (64.30%)	0.020
Diuretics	67 (9.72%)	77 (11.18%)	73 (10.60%)	0.676
Aspirins	247 (35.85%)	240 (34.83%)	254 (36.87%)	0.242
Statins	235 (34.11%)	251 (36.43%)	241 (34.99%)	0.746
Antidiabetic drugs	53 (7.69%)	87 (12.63%)	116 (16.84%)	<0.001
OSA therapy, *n* (%)				0.305
Untreated	650 (94.34%)	630 (91.44%)	634 (92.02%)	
Regular oral appliance treatment	18 (2.61%)	29 (4.21%)	26 (3.77%)	
Regular CPAP treatment	21 (3.05%)	30 (4.35%)	29 (4.21%)	

Values of continuous variables are expressed as medians (twenty-fifth percentile–seventy-fifth percentile) or means (standard deviation). Categorical variables are expressed as *n* (%). BMI: body mass index; DBP: diastolic blood pressure; FPG: fasting plasma glucose; SBP: systolic blood pressure; HDL-C: high-density lipoprotein cholesterol; eGFR: estimated glomerular filtration rate; LDL-C: low-density lipoprotein cholesterol; TC: total cholesterol; AHI: apnea hypopnea index; TG: triglyceride; ACEIs: angiotensin-converting enzyme inhibitors; ARBs: angiotensin receptor blockers; CCBs: calcium channel blockers; CPAP: continuous positive airway pressure; OSA: obstructive sleep apnea; CMI: cardiometabolic index.

**Table 2:** Hazard ratios (95% CI) of cardiovascular disease, coronary heart disease, and stroke, stratified by CMI.

Exposure	Model 1		Model 2		Model 3	
	(HR, 95% CI)	*P* value	(HR, 95% CI)	*P* value	(HR, 95% CI)	*P* value
*Cardiovascular disease*						
Per SD increment	1.48 (1.33, 1.65)	<0.001	1.35 (1.24, 1.47)	<0.001	1.31 (1.20, 1.43)	<0.001
Tertiles						
T1 (<0.73)	Reference		Reference		Reference	
T2 (≥0.73 to <1.21)	1.86 (1.32, 2.63)	<0.001	1.70 (1.25, 2.31)	<0.001	1.60 (1.18, 2.16)	0.003
T3 (≥1.21)	2.71 (1.87, 3.92)	<0.001	2.39 (1.78, 3.22)	<0.001	2.17 (1.63, 2.90)	<0.001
P for trend		<0.001		<0.001		<0.001
Categories						
T1-2 (<1.21)	Reference		Reference		Reference	
T3 (≥1.21)	1.73 (1.33, 2.25)	<0.001	1.77 (1.42, 2.22)	<0.001	1.68 (1.35, 2.09)	<0.001
*Coronary heart disease*						
Per SD increment	1.51 (1.32, 1.74)	<0.001	1.38 (1.24, 1.53)	<0.001	1.33 (1.20, 1.48)	<0.001
Tertiles						
T1 (<0.73)	Reference		Reference		Reference	
T2 (≥0.73 to <1.21)	2.25 (1.43, 3.54)	<0.001	2.06 (1.38, 3.08)	<0.001	1.90 (1.28, 2.83)	0.002
T3 (≥1.21)	3.35 (2.07, 5.44)	<0.001	2.96 (2.01, 4.37)	<0.001	2.62 (1.79, 3.83)	<0.001
P for trend		<0.001		<0.001		<0.001
Categories						
T1-2 (<1.21)	Reference		Reference		Reference	
T3 (≥1.21)	1.85 (1.32, 2.58)	<0.001	1.94 (1.46, 2.57)	<0.001	1.81 (1.37, 2.39)	<0.001
*Stroke*						
Per SD increment	1.44 (1.20, 1.72)	<0.001	1.31 (1.13, 1.52)	<0.001	1.27 (1.10, 1.47)	0.001
Tertiles						
T1 (<0.73)	Reference		Reference		Reference	
T2 (≥0.73 to <1.21)	1.48 (0.86, 2.55)	0.156	1.30 (0.80, 2.09)	0.285	1.23 (0.77, 1.98)	0.384
T3 (≥1.21)	2.02 (1.13, 3.63)	0.018	1.78 (1.12, 2.82)	0.015	1.64 (1.05, 2.57)	0.031
P for trend		0.017		0.013		0.029
Categories						
T1-2 (<1.21)	Reference		Reference		Reference	
T3 (≥1.21)	1.54 (1.02, 2.37)	0.041	1.55 (1.07, 2.25)	0.022	1.47 (1.02, 2.12)	0.039

Model 1: adjusted for age and sex. Model 2: adjusted for variables in model 1 plus drinking status, history of diabetes, smoking status, DBP, SBP, and BMI. Model 3: adjusted for all noncollinear variables. SD: standard deviation; HR: hazard ratio; CI: confidence interval. Other abbreviations appear in Table 1.

**Table 3:** Receiver operating characteristic analysis for possible predictors for predicting cardiovascular disease, coronary heart disease, and stroke.

Variables	AUC	Best threshold	Specificity	Sensitivity	PPV	NPV
*Cardiovascular disease*						
CMI	0.617	1.006	0.509	0.675	0.185	0.904
TC	0.520	4.965	0.707	0.344	0.163	0.867
TG	0.518	2.145	0.672	0.380	0.161	0.868
HDL-C	0.523	1.375	0.158	0.890	0.149	0.897
TG/HDL-C	0.521	2.613	0.768	0.291	0.172	0.868
LDL-C	0.538	2.525	0.478	0.617	0.163	0.883
FPG	0.508	5.095	0.582	0.470	0.156	0.870
WHtR	0.547	0.600	0.607	0.491	0.171	0.878
*Coronary heart disease*						
CMI	0.630	1.006	0.504	0.732	0.126	0.950
TC	0.545	4.795	0.634	0.459	0.109	0.923
TG	0.539	1.805	0.531	0.556	0.104	0.924
HDL-C	0.537	1.245	0.269	0.800	0.097	0.932
TG/HDL-C	0.543	1.441	0.413	0.688	0.103	0.931
LDL-C	0.556	2.525	0.478	0.673	0.112	0.937
FPG	0.535	5.095	0.583	0.520	0.108	0.927
WHtR	0.538	0.617	0.696	0.400	0.114	0.922
*Stroke*						
CMI	0.586	1.897	0.830	0.331	0.122	0.945
TC	0.524	4.645	0.444	0.653	0.078	0.947
TG	0.516	1.315	0.723	0.339	0.081	0.939
HDL-C	0.505	1.075	0.530	0.529	0.075	0.940
TG/HDL-C	0.517	1.466	0.586	0.512	0.081	0.944
LDL-C	0.500	1.845	0.158	0.909	0.072	0.961
FPG	0.539	4.975	0.500	0.605	0.080	0.946
WHtR	0.519	0.590	0.510	0.562	0.076	0.942

AUC: area under the receiver operating characteristic curve; NPV: negative predictive value; PPV: positive predictive value. Other abbreviations appear in Table 1.

**Table 4:** Discrimination of each predictive model for outcomes using *C*-index, continuous-NRI, and IDI.

	C-index (95% CI)	*P* value	Continuous NRI (95% CI)	*P* value	IDI (95% CI)	*P* value
*Cardiovascular disease*						
Established risk factors	0.600 (0.567, 0.633)	—	Reference	—	Reference	—
Established risk factors+CMI	0.648 (0.615, 0.681)	<0.001	0.179 (0.114, 0.248)	<0.001	0.013 (0.005, 0.025)	<0.001
*Coronary heart disease*						
Established risk factors	0.639 (0.602, 0.677)	—	Reference	—	Reference	—
Established risk factors+CMI	0.675 (0.638, 0.712)	0.001	0.173 (0.108, 0.257)	<0.001	0.008 (0.002, 0.018)	<0.001
*Stroke*						
Established risk factors	0.540 (0.484, 0.596)	—	Reference	—	Reference	—
Established risk factors+CMI	0.596 (0.541, 0.652)	0.017	0.155 (0.020, 0.272)	0.007	0.007 (0.001, 0.022)	<0.001

Established risk factors included age, sex, smoking status, drinking status, history of diabetes, BMI, TC, TG, HDL-C, LDL-C, and FPG levels. NRI: net reclassification index; IDI: integrated discrimination improvement. Other abbreviations appear in Table 1.

## Data Availability

The data used to support the findings are available from the corresponding author upon request.

## Supplementary Materials

Supplementary Materials Supplementary Methods: details of polysomnography. Supplemental Table 1: collinearity diagnostics steps. Supplemental Table 2: hazard ratios (95% CI) for cardiovascular disease, coronary heart disease, and stroke were stratified by cardiometabolic index, excluding participants who were regularly receiving CPAP treatment. Supplemental Table 3: hazard ratios (95% CI) for cardiovascular disease, coronary heart disease, and stroke stratified by cardiometabolic index, excluding current smokers. Supplemental Table 4: hazard ratios (95% CI) for cardiovascular disease, coronary heart disease, and stroke stratified by cardiometabolic index, excluding current drinkers. Supplemental Table 5: hazard ratios (95% CI) for cardiovascular disease, coronary heart disease, and stroke were stratified by cardiometabolic index, excluding participants who developed cardiovascular disease within the first year of follow-up. Supplemental Table 6: competing risk models for the association of cardiometabolic index with cardiovascular disease, coronary heart disease, and stroke. Supplementary Figure 1: comparison of area under the receiver operating characteristic cures for predicting cardiovascular disease (A), coronary heart disease (B), and stroke (C).
