# Supplementary material for: Associations of the Cardiometabolic Index with the Risk of Cardiovascular Disease in Patients with Hypertension and Obstructive Sleep Apnea: Results of a Longitudinal Cohort Study
Source: Oxid Med Cell Longev. 2022 Jun 23;2022:4914791. doi: 10.1155/2022/4914791 (PMC9246614; doi:10.1155/2022/4914791)
Supplement: Supplementary Materials — Supplementary Methods: details of polysomnography. Supplemental Table 1: collinearity diagnostics steps. Supplemental Table 2: hazard ratios (95% CI) for cardiovascular disease, coronary heart disease, and stroke were stratified by cardiometabolic index, excluding participants who were regularly receiving CPAP treatment. Supplemental Table 3: hazard ratios (95% CI) for cardiovascular disease, coronary heart disease, and stroke stratified by cardiometabolic index, excluding current smokers. Supplemental Table 4: hazard ratios (95% CI) for cardiovascular disease, coronary heart disease, and stroke stratified by cardiometabolic index, excluding current drinkers. Supplemental Table 5: hazard ratios (95% CI) for cardiovascular disease, coronary heart disease, and stroke were stratified by cardiometabolic index, excluding participants who developed cardiovascular disease within the first year of follow-up. Supplemental Table 6: competing risk models for the association of cardiometabolic index with cardiovascular disease, coronary heart disease, and stroke. Supplementary Figure 1: comparison of area under the receiver operating characteristic cures for predicting cardiovascular disease (A), coronary heart disease (B), and stroke (C). [file 4914791.f1.docx]

**Supplemental Material**

**Supplementary Methods: Details of polysomnography.**

The study was conducted in an accredited sleep laboratory. In the sleep lab, the sleep schedule was regulated, with a “lights-out” time of 12:00 pm and a “lights-on” time of 8:00 am. Participants were not permitted to sleep before lights-out time or sleep past lights-on time. Polysomnography (PSG) consisted of electroencephalograms (C4-A1, O2-A1), right and left electrooculograms, electrocardiogram (ECG), submental and anterior tibial electromyograms, thoracic and abdominal respiratory movements, pulse oxygen saturation, end-tidal and transcutaneous carbon dioxide, oronasal airflow, and nasal pressure. Studies were recorded on an E-series Sleep System (Compumedics, Melbourne, VIC, Australia) and included digital video and audio monitoring.

Studies were scored at a centralized sleep reading center (People’s Hospital of Xinjiang Uygur Autonomous Region, Urumqi, Xinjiang, China) by registered polysomnographic technologists. Sleep and arousals were staged using standard criteria ^1^. Sleep stages were scored using 30-s epochs according to Rechtschaffen and Kales criteria ^2, 3^, and sleep architecture variables were noted as percent total sleep time for stages NREM 1, NREM 2, NREM 3, and REM sleep. Arousals were scored according to published guidelines and summarized as the total number of arousals per hour of sleep (arousal index) ^4^.

To enable the calculation of alternative definitions of the apnea-hypopnea index (AHI), respiratory events were initially identified by changes in signal amplitude only. Specifically, apneas were identified as complete reductions in the thermocouple signal for ≥10 seconds and hypopneas as approximately 30% reductions in the nasal pressure or inductance signals for ≥10 seconds. Each event was annotated as to its associated oxygen saturation nadir and whether it resulted in an EEG arousal.

**References**

1. Berry RB, Budhiraja R, Gottlieb DJ, et al. Rules for scoring respiratory events in sleep: update of the 2007 AASM Manual for the Scoring of Sleep and Associated Events. Deliberations of the Sleep Apnea Definitions Task Force of the American Academy of Sleep Medicine. *J Clin Sleep Med*. 2012;8:597-619.

2. Rechtschaffen A, Kales A (1968) A manual of standardized terminology, techniques and scoring system for sleep stages of human subjects. National Institutes of Health, Washington DC.

3. Iber C, Ancoli-Israel S, Chesson A (2007) The AASM manual for the scoring of sleep and associated events: rules, terminology, and technical specifications, 1st edn. American Academy of Sleep Medicine, Westchester.

4. Amerian Academy of Sleep Medicine (1992). EEG arousals: scoring rules and examples: a preliminary report from the Sleep Disorders Atlas Task Force of the American Sleep Disorders Association. Sleep, 15(2), 173-84.

**Supplemental Table 1.** Collinearity diagnostics steps.

**Supplemental Table 2.** Hazard ratios (95% CI) for cardiovascular disease, coronary heart disease, and stroke were stratified by cardiometabolic index, excluding participants who were regularly receiving CPAP treatment.

**Supplemental Table 3.** Hazard ratios (95% CI) for cardiovascular disease, coronary heart disease, and stroke stratified by cardiometabolic index, excluding current smokers.

**Supplemental Table 4.** Hazard ratios (95% CI) for cardiovascular disease, coronary heart disease, and stroke stratified by cardiometabolic index, excluding current drinkers.

**Supplemental Table 5.** Hazard ratios (95% CI) for cardiovascular disease, coronary heart disease, and stroke were stratified by cardiometabolic index, excluding participants who developed cardiovascular disease within the first year of follow-up.

**Supplemental Table 6.** Competing risk models for the association of cardiometabolic index with cardiovascular disease, coronary heart disease, and stroke.

**Supplementary Figure 1.** Comparison of area under the receiver operating characteristic cures for predicting cardiovascular disease (A), coronary heart disease (B), and stroke (C).

**Supplemental Table 1.** Collinearity diagnostics steps.

|  | Step 1 | Step 2 |
| --- | --- | --- |
| CMI | 10.8 | 1.6 |
| Sex | 2.3 | 2.2 |
| Age | 1.5 | 1.5 |
| History of diabetes | 1.7 | 1.7 |
| Smoking status | 1.6 | 1.6 |
| Drinking status | 1.5 | 1.5 |
| BMI | 5.1 | NA |
| SBP | 2.2 | 2.2 |
| DBP | 2.4 | 2.4 |
| eGFR | 1.7 | 1.7 |
| TC | 1.9 | 1.8 |
| TG | 8.6 | NA |
| HDL-C | 2.3 | 1.7 |
| LDL-C | 1.7 | 1.7 |
| FPG | 1.7 | 1.7 |
| AHI | 1.2 | 1.2 |
| ACEIs/ARBs | 1.0 | 1.0 |
| β-Blockers | 1.0 | 1.0 |
| CCBs | 1.0 | 1.0 |
| Diuretics | 1.0 | 1.0 |
| Aspirins | 1.0 | 1.0 |
| Statins | 1.0 | 1.0 |
| Antidiabetic drugs | 1.5 | 1.5 |
| OSA therapy | 1.1 | 1.1 |

VIF: variance inflation factors.

VIF = 1/(1-R^2^). VIF step-by-step screening method: Calculate the VIF of each variable. If the maximum VIF value ≥5, remove the variable with the maximum VIF value.

**Supplemental Table 2.** Hazard ratios (95% CI) for cardiovascular disease, coronary heart disease, and stroke were stratified by cardiometabolic index, excluding participants who were regularly receiving CPAP treatment.

| Exposure | Model 1 | | Model 2 | | Model 3 | |
| --- | --- | --- | --- | --- | --- | --- |
|  | (HR, 95% CI) | P-value | (HR, 95% CI) | P-value | (HR, 95% CI) | P-value |
| **Cardiovascular disease** |  |  |  |  |  |  |
| Per SD increment | 1.49 (1.33, 1.67) | <0.001 | 1.37 (1.25, 1.49) | <0.001 | 1.33 (1.21, 1.45) | <0.001 |
| Tertiles |  |  |  |  |  |  |
| T1 | Reference |  | Reference |  | Reference |  |
| T2 | 1.91 (1.33, 2.74) | 0.001 | 1.73 (1.26, 2.39) | 0.001 | 1.63 (1.18, 2.23) | 0.003 |
| T3 | 2.84 (1.93, 4.18) | <0.001 | 2.54 (1.87, 3.46) | <0.001 | 2.29 (1.70, 3.10) | <0.001 |
| Categories |  |  |  |  |  |  |
| T1-2 | Reference |  | Reference |  | Reference |  |
| T3 | 1.78 (1.36, 2.34) | <0.001 | 1.86 (1.47, 2.36) | <0.001 | 1.75 (1.39, 2.21) | <0.001 |
| **Coronary heart disease** |  |  |  |  |  |  |
| Per SD increment | 1.51 (1.30, 1.74) | <0.001 | 1.38 (1.23, 1.54) | <0.001 | 1.33 (1.20, 1.49) | <0.001 |
| Tertiles |  |  |  |  |  |  |
| T1 | Reference |  | Reference |  | Reference |  |
| T2 | 2.26 (1.42, 3.61) | 0.001 | 2.07 (1.37, 3.13) | 0.001 | 1.91 (1.27, 2.88) | 0.002 |
| T3 | 3.36 (2.04, 5.54) | <0.001 | 3.01 (2.02, 4.50) | <0.001 | 2.66 (1.80, 3.94) | <0.001 |
| Categories |  |  |  |  |  |  |
| T1-2 | Reference |  | Reference |  | Reference |  |
| T3 | 1.85 (1.31, 2.61) | 0.001 | 1.97 (1.47, 2.64) | <0.001 | 1.84 (1.38, 2.45) | <0.001 |
| **Stroke** |  |  |  |  |  |  |
| Per SD increment | 1.49 (1.24, 1.79) | <0.001 | 1.35 (1.16, 1.57) | <0.001 | 1.31 (1.13, 1.52) | <0.001 |
| Tertiles |  |  |  |  |  |  |
| T1 | Reference |  | Reference |  | Reference |  |
| T2 | 1.52 (0.85, 2.73) | 0.158 | 1.33 (0.80, 2.23) | 0.271 | 1.26 (0.76, 2.09) | 0.374 |
| T3 | 2.24 (1.20, 4.17) | 0.011 | 2.00 (1.23, 3.25) | 0.005 | 1.82 (1.13, 2.92) | 0.014 |
| Categories |  |  |  |  |  |  |
| T1-2 | Reference |  | Reference |  | Reference |  |
| T3 | 1.67 (1.06, 2.63) | 0.028 | 1.71 (1.16, 2.53) | 0.007 | 1.61 (1.10, 2.37) | 0.015 |

Model 1: adjusted for age and sex.

Model 2: adjusted for variables in model 1 plus drinking status, history of diabetes, smoking status, DBP, SBP, and BMI.

Model 3: adjusted for all non-collinear variables.

SD, standard deviation; HR, hazard ratio; CI, confidence interval. Other abbreviations appear in Table 1.

**Supplemental Table 3.** Hazard ratios (95% CI) for cardiovascular disease, coronary heart disease, and stroke stratified by cardiometabolic index, excluding current smokers.

| Exposure | Model 1 | | Model 2 | | Model 3 | |
| --- | --- | --- | --- | --- | --- | --- |
|  | (HR, 95% CI) | P-value | (HR, 95% CI) | P-value | (HR, 95% CI) | P-value |
| **Cardiovascular disease** |  |  |  |  |  |  |
| Per SD increment | 1.51 (1.32, 1.72) | <0.001 | 1.41 (1.27, 1.57) | <0.001 | 1.37 (1.23, 1.52) | <0.001 |
| Tertiles |  |  |  |  |  |  |
| T1 | Reference |  | Reference |  | Reference |  |
| T2 | 1.96 (1.30, 2.96) | 0.001 | 1.78 (1.25, 2.53) | 0.001 | 1.70 (1.20, 2.41) | 0.003 |
| T3 | 2.54 (1.62, 4.00) | <0.001 | 2.23 (1.57, 3.17) | <0.001 | 2.09 (1.48, 2.95) | <0.001 |
| Categories |  |  |  |  |  |  |
| T1-2 | Reference |  | Reference |  | Reference |  |
| T3 | 1.57 (1.13, 2.19) | 0.007 | 1.66 (1.25, 2.20) | 0.001 | 1.59 (1.21, 2.11) | 0.001 |
| **Coronary heart disease** |  |  |  |  |  |  |
| Per SD increment | 1.52 (1.28, 1.80) | <0.001 | 1.42 (1.23, 1.63) | <0.001 | 1.36 (1.18, 1.56) | <0.001 |
| Tertiles |  |  |  |  |  |  |
| T1 | Reference |  | Reference |  | Reference |  |
| T2 | 2.35 (1.37, 4.05) | 0.002 | 2.07 (1.30, 3.27) | 0.002 | 1.92 (1.21, 3.03) | 0.005 |
| T3 | 2.96 (1.63, 5.40) | <0.001 | 2.48 (1.56, 3.95) | <0.001 | 2.24 (1.42, 3.54) | 0.001 |
| Categories |  |  |  |  |  |  |
| T1-2 | Reference |  | Reference |  | Reference |  |
| T3 | 1.60 (1.04, 2.46) | 0.034 | 1.69 (1.17, 2.44) | 0.005 | 1.59 (1.11, 2.29) | 0.011 |
| **Stroke** |  |  |  |  |  |  |
| Per SD increment | 1.53 (1.24, 1.88) | <0.001 | 1.41 (1.19, 1.67) | <0.001 | 1.39 (1.17, 1.64) | 0.0001 |
| Tertiles |  |  |  |  |  |  |
| T1 | Reference |  | Reference |  | Reference |  |
| T2 | 1.64 (0.87, 3.09) | 0.125 | 1.46 (0.85, 2.53) | 0.174 | 1.43 (0.83, 2.47) | 0.196 |
| T3 | 2.19 (1.09, 4.38) | 0.027 | 1.97 (1.15, 3.37) | 0.014 | 1.90 (1.12, 3.22) | 0.018 |
| Categories |  |  |  |  |  |  |
| T1-2 | Reference |  | Reference |  | Reference |  |
| T3 | 1.55 (0.92, 2.60) | 0.099 | 1.63 (1.04, 2.54) | 0.033 | 1.59 (1.02, 2.47) | 0.039 |

Model 1: adjusted for age and sex.

Model 2: adjusted for variables in model 1 plus drinking status, history of diabetes, smoking status, DBP, SBP, and BMI.

Model 3: adjusted for all non-collinear variables.

SD, standard deviation; HR, hazard ratio; CI, confidence interval. Other abbreviations appear in Table 1.

**Supplemental Table 4.** Hazard ratios (95% CI) for cardiovascular disease, coronary heart disease, and stroke stratified by cardiometabolic index, excluding current drinkers.

| Exposure | Model 1 | | Model 2 | | Model 3 | |
| --- | --- | --- | --- | --- | --- | --- |
|  | (HR, 95% CI) | P-value | (HR, 95% CI) | P-value | (HR, 95% CI) | P-value |
| **Cardiovascular disease** |  |  |  |  |  |  |
| Per SD increment | 1.58 (1.39, 1.80) | <0.001 | 1.44 (1.30, 1.60) | <0.001 | 1.43 (1.29, 1.58) | <0.001 |
| Tertiles |  |  |  |  |  |  |
| T1 | Reference |  | Reference |  | Reference |  |
| T2 | 2.16 (1.44, 3.25) | <0.001 | 1.97 (1.38, 2.80) | <0.001 | 1.90 (1.34, 2.70) | <0.001 |
| T3 | 2.79 (1.79, 4.35) | <0.001 | 2.49 (1.75, 3.53) | <0.001 | 2.37 (1.68, 3.33) | <0.001 |
| Categories |  |  |  |  |  |  |
| T1-2 | Reference |  | Reference |  | Reference |  |
| T3 | 1.60 (1.16, 2.20) | 0.004 | 1.72 (1.31, 2.25) | <0.001 | 1.68 (1.28, 2.19) | <0.001 |
| **Coronary heart disease** |  |  |  |  |  |  |
| Per SD increment | 1.53 (1.29, 1.82) | <0.001 | 1.44 (1.26, 1.64) | <0.001 | 1.42 (1.24, 1.61) | <0.001 |
| Tertiles |  |  |  |  |  |  |
| T1 | Reference |  | Reference |  | Reference |  |
| T2 | 2.22 (1.31, 3.74) | 0.003 | 2.14 (1.36, 3.37) | 0.001 | 2.03 (1.29, 3.18) | 0.002 |
| T3 | 2.75 (1.55, 4.88) | 0.001 | 2.67 (1.70, 4.20) | <0.001 | 2.48 (1.59, 3.86) | <0.001 |
| Categories |  |  |  |  |  |  |
| T1-2 | Reference |  | Reference |  | Reference |  |
| T3 | 1.54 (1.02, 2.31) | 0.039 | 1.75 (1.24, 2.48) | 0.002 | 1.69 (1.20, 2.37) | 0.003 |
| **Stroke** |  |  |  |  |  |  |
| Per SD increment | 1.66 (1.36, 2.03) | <0.001 | 1.47 (1.25, 1.73) | <0.001 | 1.45 (1.23, 1.70) | <0.001 |
| Tertiles |  |  |  |  |  |  |
| T1 | Reference |  | Reference |  | Reference |  |
| T2 | 2.25 (1.17, 4.34) | 0.016 | 1.76 (1.00, 3.08) | 0.049 | 1.72 (0.99, 3.01) | 0.056 |
| T3 | 2.99 (1.47, 6.08) | 0.003 | 2.29 (1.32, 3.97) | 0.003 | 2.20 (1.28, 3.79) | 0.004 |
| Categories |  |  |  |  |  |  |
| T1-2 | Reference |  | Reference |  | Reference |  |
| T3 | 1.68 (1.01, 2.80) | 0.045 | 1.69 (1.09, 2.62) | 0.019 | 1.66 (1.08, 2.55) | 0.021 |

Model 1: adjusted for age and sex.

Model 2: adjusted for variables in model 1 plus drinking status, history of diabetes, smoking status, DBP, SBP, and BMI.

Model 3: adjusted for all non-collinear variables.

SD, standard deviation; HR, hazard ratio; CI, confidence interval. Other abbreviations appear in Table 1.

**Supplemental Table 5.** Hazard ratios (95% CI) for cardiovascular disease, coronary heart disease, and stroke were stratified by cardiometabolic index, excluding participants who developed cardiovascular disease within the first year of follow-up.

| Exposure | Model 1 | | Model 2 | | Model 3 | |
| --- | --- | --- | --- | --- | --- | --- |
|  | (HR, 95% CI) | P-value | (HR, 95% CI) | P-value | (HR, 95% CI) | P-value |
| **Cardiovascular disease** |  |  |  |  |  |  |
| Per SD increment | 1.52 (1.36, 1.69) | <0.001 | 1.36 (1.25, 1.49) | <0.001 | 1.32 (1.21, 1.44) | <0.001 |
| Tertiles |  |  |  |  |  |  |
| T1 | Reference |  | Reference |  | Reference |  |
| T2 | 1.82 (1.28, 2.59) | 0.001 | 1.66 (1.21, 2.27) | 0.002 | 1.56 (1.14, 2.14) | 0.005 |
| T3 | 2.77 (1.90, 4.05) | <0.001 | 2.39 (1.76, 3.23) | <0.001 | 2.17 (1.61, 2.91) | <0.001 |
| Categories |  |  |  |  |  |  |
| T1-2 | Reference |  | Reference |  | Reference |  |
| T3 | 1.81 (1.38, 2.37) | <0.001 | 1.79 (1.42, 2.26) | <0.001 | 1.70 (1.35, 2.13) | <0.001 |
| **Coronary heart disease** |  |  |  |  |  |  |
| Per SD increment | 1.56 (1.35, 1.79) | <0.001 | 1.40 (1.25, 1.56) | <0.001 | 1.36 (1.22, 1.51) | <0.001 |
| Tertiles |  |  |  |  |  |  |
| T1 | Reference |  | Reference |  | Reference |  |
| T2 | 2.13 (1.33, 3.39) | 0.002 | 1.98 (1.31, 3.00) | 0.001 | 1.84 (1.22, 2.78) | 0.004 |
| T3 | 3.38 (2.05, 5.56) | <0.001 | 2.94 (1.97, 4.39) | <0.001 | 2.63 (1.78, 3.89) | <0.001 |
| Categories |  |  |  |  |  |  |
| T1-2 | Reference |  | Reference |  | Reference |  |
| T3 | 1.95 (1.38, 2.74) | <0.001 | 1.98 (1.48, 2.65) | <0.001 | 1.86 (1.39, 2.47) | <0.001 |
| **Stroke** |  |  |  |  |  |  |
| Per SD increment | 1.46 (1.22, 1.75) | <0.001 | 1.30 (1.12, 1.52) | 0.001 | 1.26 (1.08, 1.46) | 0.003 |
| Tertiles |  |  |  |  |  |  |
| T1 | Reference |  | Reference |  | Reference |  |
| T2 | 1.54 (0.88, 2.67) | 0.128 | 1.32 (0.81, 2.14) | 0.265 | 1.24 (0.77, 2.01) | 0.377 |
| T3 | 2.15 (1.19, 3.91) | 0.012 | 1.79 (1.12, 2.87) | 0.015 | 1.63 (1.03, 2.58) | 0.037 |
| Categories |  |  |  |  |  |  |
| T1-2 | Reference |  | Reference |  | Reference |  |
| T3 | 1.60 (1.03, 2.48) | 0.038 | 1.55 (1.06, 2.26) | 0.025 | 1.46 (1.00, 2.11) | 0.048 |

Model 1: adjusted for age and sex.

Model 2: adjusted for variables in model 1 plus drinking status, history of diabetes, smoking status, DBP, SBP, and BMI.

Model 3: adjusted for all non-collinear variables.

SD, standard deviation; HR, hazard ratio; CI, confidence interval. Other abbreviations appear in Table 1.

**Supplemental Table 6.** Competing risk models for the association of cardiometabolic index with cardiovascular disease, coronary heart disease, and stroke.

| Exposure | Model 1 | | Model 2 | | Model 3 | |
| --- | --- | --- | --- | --- | --- | --- |
|  | (SHR, 95% CI) | P-value | (SHR, 95% CI) | P-value | (SHR, 95% CI) | P-value |
| **Cardiovascular disease** |  |  |  |  |  |  |
| Per SD increment | 1.42 (1.26, 1.61) | <0.001 | 1.32 (1.19, 1.46) | <0.001 | 1.28 (1.16, 1.41) | <0.001 |
| Tertiles |  |  |  |  |  |  |
| T1 | Reference |  | Reference |  | Reference |  |
| T2 | 1.75 (1.20, 2.56) | 0.004 | 1.60 (1.14, 2.25) | 0.007 | 1.51 (1.07, 2.11) | 0.017 |
| T3 | 2.39 (1.60, 3.56) | <0.001 | 2.14 (1.54, 2.96) | <0.001 | 1.96 (1.42, 2.69) | <0.001 |
| Categories |  |  |  |  |  |  |
| T1-2 | Reference |  | Reference |  | Reference |  |
| T3 | 1.63 (1.22, 2.18) | 0.001 | 1.64 (1.28, 2.11) | <0.001 | 1.56 (1.23, 2.00) | <0.001 |
| **Coronary heart disease** |  |  |  |  |  |  |
| Per SD increment | 1.42 (1.21, 1.66) | <0.001 | 1.32 (1.16, 1.50) | <0.001 | 1.26 (1.12, 1.43) | <0.001 |
| Tertiles |  |  |  |  |  |  |
| T1 | Reference |  | Reference |  | Reference |  |
| T2 | 2.10 (1.28, 3.42) | 0.003 | 2.10 (1.35, 3.26) | 0.001 | 1.89 (1.22, 2.91) | 0.004 |
| T3 | 2.70 (1.60, 4.55) | <0.001 | 2.58 (1.68, 3.98) | <0.001 | 2.24 (1.47, 3.40) | <0.001 |
| Categories |  |  |  |  |  |  |
| T1-2 | Reference |  | Reference |  | Reference |  |
| T3 | 1.60 (1.11, 2.29) | 0.011 | 1.67 (1.22, 2.29) | 0.001 | 1.55 (1.14, 2.11) | 0.005 |
| **Stroke** |  |  |  |  |  |  |
| Per SD increment | 1.46 (1.20, 1.78) | <0.001 | 1.31 (1.11, 1.55) | 0.001 | 1.30 (1.11, 1.53) | 0.001 |
| Tertiles |  |  |  |  |  |  |
| T1 | Reference |  | Reference |  | Reference |  |
| T2 | 1.38 (0.75, 2.53) | 0.299 | 1.05 (0.61, 1.81) | 0.866 | 1.04 (0.60, 1.79) | 0.891 |
| T3 | 2.00 (1.07, 3.75) | 0.031 | 1.65 (0.99, 2.73) | 0.053 | 1.61 (0.98, 2.63) | 0.058 |
| Categories |  |  |  |  |  |  |
| T1-2 | Reference |  | Reference |  | Reference |  |
| T3 | 1.65 (1.02, 2.67) | 0.042 | 1.61 (1.07, 2.44) | 0.023 | 1.58 (1.06, 2.37) | 0.025 |

Model 1: adjusted for age and sex.

Model 2: adjusted for variables in model 1 plus drinking status, history of diabetes, smoking status, DBP, SBP, and BMI.

Model 3: adjusted for all non-collinear variables.

SD, standard deviation; SHR, sub-distribution hazard ratio; CI, confidence interval. Other abbreviations appear in Table 1.


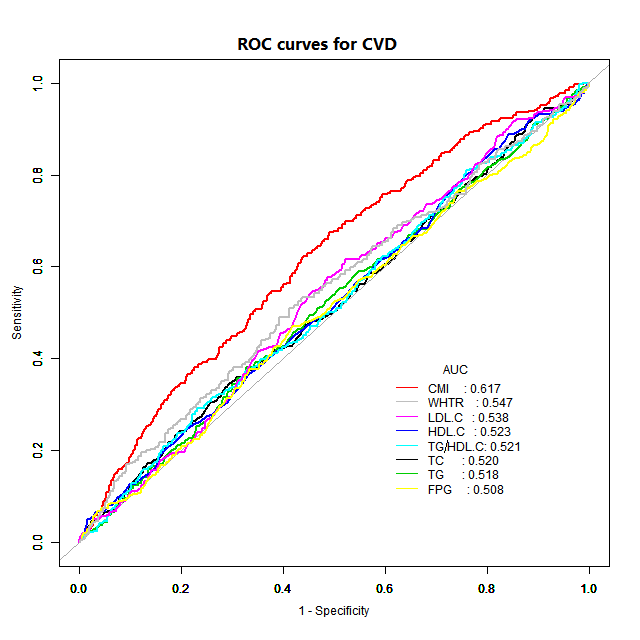


**Supplementary Figure 1A**


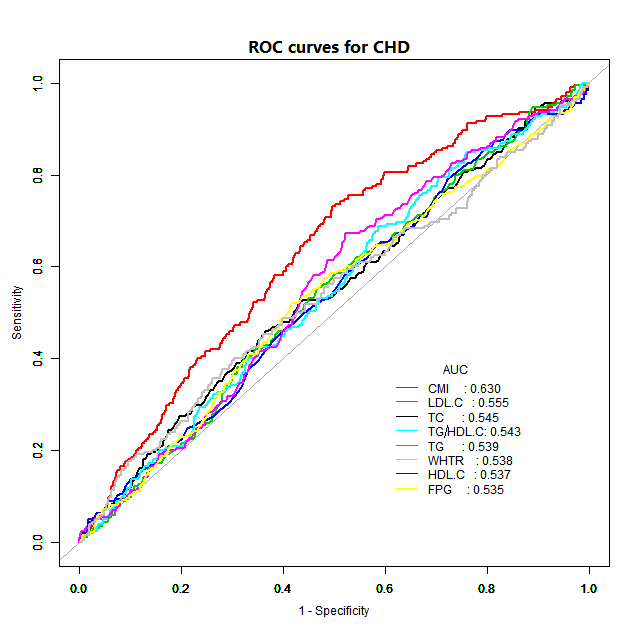


**Supplementary Figure 1B**


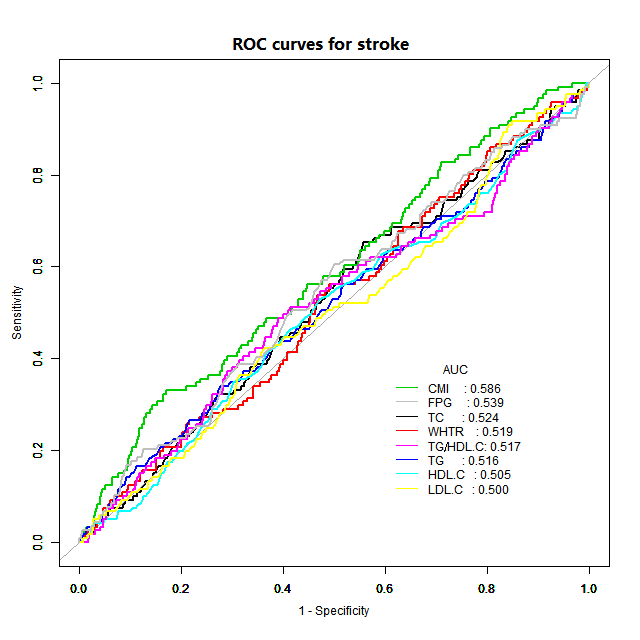


**Supplementary Figure 1C**
